# Resistant effects determination of *Lactobacillus* supplementation on broilers to consecutive hydrogen sulfide exposure

**DOI:** 10.1016/j.psj.2023.103102

**Published:** 2023-09-11

**Authors:** Xiao Zhou, Meijun Hu, Jiahui Luo, Binghong Xie, Pengyun Ma, Guoyun Wu, Fuguang Xue

**Affiliations:** ⁎ School of Mathematics, Physics and Optoelectronic Engineering, Hubei University of Automotive Technology, Shiyan, Hubei 442002, China; † Nanchang key laboratory of animal health and safety production, Jiangxi Agricultural University, Nanchang, Jiangxi 330045, China

**Keywords:** hydrogen sulfide, *Lactobacillus,* broilers, gut microbiome, physiological metabolomics

## Abstract

Hydrogen sulfide (**H_2_S**) is one of the most irritant gases present in rearing stalls that suppress broilers’ healthy growth, which is seriously required an effective alleviation method. In this study, *Lactobacillus* was supplemented to investigate the alleviative effects on broilers reared under consecutive H_2_S exposure. A total of 180 healthy 1-day-old male AA broilers with similar body weight (40.8 ± 1.0 g) were randomly allotted into the control treatment (**CON**), the hydrogen sulfide treatment (H_2_S), and the *Lactobacillus* supplement under H_2_S exposure treatment (**LAC**) for a 42-d-long feeding process. Growth and carcass performances, immunity-related parameters, intestinal development and cecal microbial communities, and blood metabolites were measured. Results showed that *Lactobacillus* supplement significantly increased the body weight gain (**BWG**) while reduced the mortality rate, abdominal fat and bursa of fabricius weight during the whole rearing time compared with H_2_S treatment (*P <* 0.05). Serum LPS, IL-1β, IL-2, and IL-6 contents were observed significantly increased after H_2_S treatment while remarkably decreased after *Lactobacillus* supplementation(*P* < 0.05). Intestinal morphology results showed a significant higher in the development of ileum villus height (*P* < 0.05). Cecal microbiota results showed the bacterial composition was significantly altered after *Lactobacillus* supplement (*P* < 0.05). Specifically, *Lactobacillus* supplement significantly decreased the relative abundance of *Faecalibacterium,* while significantly proliferated the relative abundance of *Lactobacillus, Bifidobacterium, Clostridium, and Campylobacter* (*P*<0.05). Metabolic results indicated that *Lactobacillus* supplement may alleviate the harmful effects caused by H_2_S through regulating the pyrimidine metabolism, starch and sucrose metabolism, fructose and mannose degradation, and beta-alanine metabolism. In summary, *Lactobacillus* supplement effectively increased BWG and decreased mortality rate of broilers under H_2_S exposure by enhancing the body's immune capacity, proliferating beneficial microbes (e.g., *Lactobacillus* and *Bifidobacterium*), and regulating the physiological pyrimidine metabolism, starch and sucrose metabolism, and beta-alanine metabolism.

## INTRODUCTION

The environmental comfort of animals, particularly in relation to harmful gas content, seriously impact the growth performance, management, and economic benefits of broiler production. Among these gases, hydrogen sulfide (**H_2_S**) is one of the most common irritants present in the animal growth environment. Its presence can lead to various detrimental effects on the animals, including loss of appetite (Kafle and Chen, 2014), cellular damage, and inflammatory responses (Wang et al., 2019). Furthermore, H_2_S has been found to suppress the expression of energy metabolism-related genes (Untereiner et al., 2017; Wang et al., 2019; Yang et al., 2019), significantly decreasing the provision of physiological energy and disrupting the host's physiological homeostasis. Prolonged exposure to H_2_S also triggers apoptosis in the respiratory tract and intestinal epithelium, inhibits the transportation of essential nutrients, and hinders growth performance (Wetzel and Wenke, 2019). Therefore, it is imperative to implement proper strategies that effectively alleviate the detriments caused by H_2_S inhalation.

The gut microbiome was shown effectively impacted the phenotypic outcomes through interactions with host genes (Jackrel et al., 2020). Alterations in intestinal microbiome communities primarily acted on nutrient degradability, impacting physiological metabolic processes, altering the growth performance and immunity of broilers. Probiotics such as *Lactobacillus* co-evolved with the host immune system, and effectively promote innate and adaptive immune responses (O'Callaghan and O'Toole, 2013). Further, previous study indicated the beneficial effects of proper supplementation of *Lactobacillus* on nutrient absorption (Wang et al., 2021) and antioxidative capacity, and reduced inflammatory response (Nam et al., 2022). Besides, *Lactobacillus* supplement also improved intestinal health (Li et al., 2022), enhanced humoral immunity (Sun et al., 2022), and boosted the growth performance of broilers. However, limited information is available on whether *Lactobacillus* supplementation helps alleviate the negative effects of H_2_S inhalation.

In this study, we supplemented *Lactobacillus* in the broiler diet to examine its potential alleviating effects on broilers exposed to H_2_S. We utilized metabolomics and 16S rRNA sequencing methods to investigate the nutritional metabolic and microbial alterations induced by *Lactobacillus* supplementation in order to determine the potential protective effects of *Lactobacillus* supplementation against H_2_S exposure.

## MATERIALS AND METHODS

Animal care and procedures followed The Chinese Guidelines for Animal Welfare, which was approved by the Animal Care and Use Committee of Jiangxi Agricultural University. The approval number is JXAULL-20230126.

### Experimental Design and Treatment Process

A total of 180 healthy 1-day-old male AA broilers with similar body weight (40.8±1.0 g) were randomly allotted into the control treatment (**CON**), the hydrogen sulfide exposure treatment (H_2_S), and the *Lactobacillus* supplement under H_2_S exposure treatment (**LAC**). Each treatment contained 6 replicates, with 10 birds in each replicate. All broilers were reared in battery pens (100 cm × 70 cm) with plastic wire floors in the environmentally controlled chambers of Jiangxi Agricultural University. All birds were provided a 2-phases feeding program (D 0-21 as phase 1, D 22-42 as phase 2), and allowed with the feed and water *ad libitum* throughout the experiment. The composition of the experimental diets and the nutrients of both phase 1 and phase 2 were shown in Table 1. *Lactobacillus spp.* strains which including *Lactobacillus acidophilus, Lactobacillus salivarius and Lactobacillus agilis* for animal feed additive was provided by Shandong Fuhe Biotechnology Co. LTD (Jinan, Shandong Province, China) with an effective bacterial count of approximately 1  ×  10^8^ CFU/g, and supplemented 1g/d per bird by the formation of microencapsulation into the premix based on the previous study of Hong et al. (2021). The concentration of H_2_S in both H_2_S and LAC treatments were set at 30 ppm based on the previous research of Wang et al. (2019), while the H_2_S concentration in CON was kept below 5ppm. Room temperature was maintained at 37°C for the first 3 d and gradually reduced to 33°C in the first week. Following, the temperature will decrease by 3°C each week until reaching 24°C and the lighting schedule was set with 23 h light and 1 h dark during the experiment periods.

**Table 1:** Composition of the experimental diets for broilers.

Ingredient	1–21 d	22–42 d
Corn	59.7	60.4
soy oil	1.45	2.98
Soybean Meal (CP 43%)	34.6	32.68
L- Lys-HCL (98%)	0.17	0.18
DL-Met	0.24	0.23
CaCO_3_	1.2	1
Calcium hydrophosphate	1.86	1.8
Salt	0.4	0.4
Choline HCl (50%)	0.15	0.1
Primix Vitamin^1^	0.03	0.03
Primix mineral^2^	0.2	0.2
Total	100	100
Level of nutrients (calculated)		
ME/(kcal/kg)	2,950	3,020
CP	21	20
Ca	1.01	0.9
P	0.45	0.43
dLys	1.15	1.1
dMet	0.5	0.48
dCys	0.29	0.28
dM+C	0.86	0.82

1 Vitamin content: VA12000IU/kg; VD_3_3000IU/kg; VE7.5IU/kg; VK_3_1.50 mg/kg; VB_1_ 0.6 mg/kg; VB_2_ 4.8 mg/kg; VB_6_ 1.8 mg/kg; VB_12_ 10 mg/kg; Folic acid 0.15 mg/kg; niacinamide 30 mg/kg; pantothenic acid 10.5 mg/kg.

2 Fe 80 mg, Cu 8 mg, Mn 80 mg, Zn 60 mg, Se 0.15 mg, I 0.35 mg.

### Growth and Carcass Performances Measurement

The body weight of each broiler was recorded at the hatching day, d 21, and d 42, respectively, followed by the calculation of the body weight gain (**BWG**) in each phase on the basis of replicate. The feed intake (**FI**) was recorded every day based on the deviation between the residue and the provision. The feed conversion ratio (**FCR**) between feed intake and body weight gain was calculated based on the following equation,

FCR=feedintake(g)/bodyweightgain(g)

Carcass characteristics were following measured based on the randomly selected 3 birds per replication (total of 48 birds) on the last day of the experiment. Dressing percentage, eviscerated yield, breast muscle, thigh muscle, and abdominal fat are separated and weighed. The yielding rate of eviscerated weight, breast, thigh, and abdominal were calculated according to the methods described in our previous study (Xue et al., 2020). The immune organs including the spleen, thymus gland, and bursa of Fabricius were also weighed, respectively.

### Serum Immunity-Related Parameters Measurement

Blood samples were harvested from the above-mentioned 48 birds by wing vein blood collection method, each bird was collected with 5 milliliters. Serum was following acquired by centrifuging at room temperature at 3,000 *g* for 10 min. Concentrations of serum immune globulins which included IgA, IgM, and IgG, interleukin factors such as IL-1, IL-2, IL-4, and IL-6, and the lipopolysaccharide (**LPS**) content were assayed using chicken ELISA quantitation Kits (Nanjing, Jiangsu Province, China).

### Blood Metabolomic Measurements

The LC/MS analyzing method was applied in our present study for the measurement of blood metabolic responses to *Lactobacillus* supplement under consecutive H_2_S exposure treatment. To be simply stated, the internal standard was firstly prepared by dissolving 100 µL blood with 400 µL mixed liquor, which consisted of 80% methanol solution and 0.02 mg/mL L-2-chlorophenyl alanine. All samples were subsequently cleaned using ultrasound at 40 kHz and 5°C for 30 min, centrifuged with 13,000 *g* at 4°C for 15 min, and carefully transferred to sample vials for LC-MS/MS analysis. Chromatographic separation of the metabolites was conducted using the Thermo UHPLC system which was equipped with an ACQUITY UPLC HSS T3 (100 mm × 2.1 mm i.d., 1.8 µm; Waters, Milford, CT), followed by the mass spectrometric data collection by Thermo UHPLC-Q Exactive HF-X Mass Spectrometer equipped with electrospray ionization (ESI) source in either positive or negative ion mode.

### Morphological Examination of the Intestinal Wall

The paraffin section of both ileum and jejunum was made for the intestinal morphological parameter measurement, which included the villus height and crypt depth of each intestinal segment. Villus height and crypt depth were calculated as the average of 10 villi and crypts to avoid random errors. The villus height to crypt depth ratio was further calculated based on the above-calculated results.

### Cecal Content Sampling and Microbiota Analysis

The cecal contents of each bird were sampled for the microbial diversity measurement through 16S rRNA sequencing method. Briefly stated, the cetyltrimethylammonium bromide and sodium dodecyl sulfate (**CTAB/SDS**) method (Xue et al., 2020) was applied for DNA extraction, followed by the design of the primers based on the 16S rRNA V4 region. Primers of 520F (F: GTGCCAGCMGCCGCGGTAA) and 802R (R: GGACTACHVGGGTWTCTAAT) were chosen for the PCR amplification process. TruSeq® DNA PCR-Free Sample Preparation Kit (Illumina Inc., San Diego, CA) was used for the sequencing library establishment, while the Qubit@ 2.0 Fluorometer (Thermo Scientific) and Agilent Bioanalyzer 2100 system were applied for the library quality assessment. PCR amplification output of each sample was sequenced using Illumina HiSeq 4000 platform (Illumina Inc., San Diego, CA), and the quality of raw tags was filtered by Quantitative Insights Into Microbial Ecology (QIIME, V1.7.0). Sequences within similarity >97% were assigned to the same operational taxonomic unit (**OTU**), and subsequently, GreenGene Database was applied to annotate taxonomic information of all filtered OTUs.

### Statistical Analysis

Normal distribution test using the SAS (Statistics Analysis System, version 9.4, SAS Institute Inc., Cary, NC) procedure “proc univariate data=test normal” was firstly conducted on the growth performances, carcass performances, serum immunity-related parameters, and gastrointestinal morphology parameters, followed by the differential analysis using the 1-way ANOVA S-N-K test among 3 treatments. Results were presented as mean ± SEM. *P*-value < 0.05 was considered to be significant, and 0.05 ≤ *P* < 0.10 was considered a tendency.

As mentioned in the term microbial diversity analysis, OTU abundances of each sample were first conducted a normal distribution testing using the SAS procedure “proc univariate data=test normal,” followed by the differential analysis through the 1-way ANOVA S-N-K test. Differential analysis on the alpha diversity and beta diversity of each treatment was primarily calculated with QIIME (version 1.7.0) and displayed with R software (version 4.1.3, R Core Team, Vienna, Austria). Principal coordinate analysis (**PCoA**) was applied to determine the discrepancies among all 3 treatments by ggplot2 package in R software. Spearman correlations between microbial communities and immunity-related parameters were assessed using the PROC CORR procedure of SAS 9.4, followed by the creation of a correlation matrix and visualization in a heatmap format using R software (version 4.1.3, R Core Team, Vienna, Austria).

As referred to the metabolomic analysis, the raw data acquisition was performed using the Data Dependent Acquisition (**DDA**) mode, followed by the importation into the Progenesis QI 2.3 (Nonlinear Dynamics, Waters) for peak detection and alignment. Results were generated into a data matrix, which consisted of the retention time (**RT**), mass-to-charge ratio (m/z) values, and peak intensity. Pathway analysis on differential metabolites was conducted by MetaboAnalyst 5.0 (https://www.metaboanalyst.ca/), and results were displayed in the formation of a bubble diagram which was calculated according to the *P*-values and impacts of each enriched pathway.

## RESULTS

### Effects of Lactobacillus Supplementation on Growth and Carcass Performances of Broilers

As shown in Table 2, In the initial phase, no significant changes were detected in FI and BWG among all 3 treatments (*P >* 0.05). In the entire phase, there was a significant decrease in BWG under the H_2_S exposure treatment compared with the control (CON) group (*P <* 0.05). However, a significant increment in BWG was observed following *Lactobacillus* supplementation. No significant alterations were observed in FI and FCR during the whole phase across all treatments (*P >* 0.05). Notably, the mortality rate significantly decreased following *Lactobacillus* supplementation compared with the H_2_S exposure treatment (*P <* 0.05).

**Table 2:** Effects of *Lactobacillus* supplement on productive performances, carcass performances, and immune organs of broilers under consecutive H_2_S treatment (n = 6).

Items	CON	H_2_S	LAC	SEM	*P*-value
FI_(21)_(g)	1,178	1,133	1,143	34.64	0.174
BWG_(21)_(g)	903.4	856.3	877.6	21.72	0.097
FI_(42)_(g)	3,972	3,877	3,949	101.2	0.147
BWG_(42)_(g)	2,456^a^	2,318^b^	2,415^a^	34.7	0.029
FCR	1.62	1.67	1.65	0.041	0.087
Mortality rate (%)	5.33^b^	6.25^a^	5.08^b^	0.23	0.019
Dressed percentage (%)	92.47	92.14	92.44	1.172	0.349
Eviscerated percentage (%)	74.43	73.88	74.53	1.684	0.665
Breast muscle rate (%)	31.29	31.37	31.34	1.342	0.296
Thigh muscle rate (%)	24.38	23.99	23.72	0.431	0.231
Abdominal fat rate (%)	1.21^a^	1.17^a^	1.08^b^	0.061	0.034
Spleen (g)	2.39	2.28	2.49	0.243	0.176
Thymus (g)	4.41	4.84	4.36	0.417	0.247
Bursa of Fabricius (g)	3.72^b^	4.29^a^	3.94^b^	0.124	0.034

Abbreviations: BWG _(21)_, body weight gain in the first phase; BWG_(42)_, body weight gain in the whole phase; CON, control treatment; FCR, feed conversion ratio; FI_(21)_, feed intake in the initial phase; FI _(42)_, feed intake in the whole phase; H_2_S, hydrogen sulfide treatment; LAC, *Lactobacillus* supplement treatment.

a,b means within a row with different letters differed significantly (*P* < 0.05).

In terms of carcass performances, the supplementation of *Lactobacillus* resulted in a significant decrease in abdominal fat compared with both the CON and H_2_S exposure groups (*P <* 0.05). No significant changes were detected in dressed percentage, eviscerated percentage, breast and thigh muscle rates (*P >* 0.05). Additionally, the weight of the bursa of Fabricius significantly increased under H_2_S exposure, but significantly decreased upon *Lactobacillus* supplementation (*P <* 0.05). No significant alterations were observed in the spleen and thymus across all treatments (*P >* 0.05).

### Effects of Lactobacillus Supplementation on Serum Immunity

The immune responses to H_2_S and *Lactobacillus* supplement treatments are presented in Table 3. Higher levels of IgA were observed in the H_2_S exposure group compared with both the CON and *Lactobacillus* supplementation (LAC) groups (*P <* 0.05). However, no significant changes were found in IgM and IgG levels (*P >* 0.05). The contents of lipopolysaccharide (LPS) and serum IL-1β, IL-2, and IL-6 significantly increased under the H_2_S exposure treatment, but notably decreased following *Lactobacillus* supplementation (*P <* 0.05). Additionally, the IL-4 content significantly declined under the H_2_S treatment compared with the CON group (*P <* 0.05), but no significant increase was observed after *Lactobacillus* supplementation (*P >* 0.05).

**Table 3:** Effects of *Lactobacillus* supplement on serum immune-related parameters of broilers under consecutive H_2_S treatment (n = 6).

Items	CON	H_2_S	LAC	SEM	*P*-value
IgM (g/L)	1.39	1.56	1.47	0.17	0.192
IgA (g/L)	2.02^b^	2.91^a^	2.23^b^	0.33	0.016
IgG (g/L)	15.21	17.29	15.26	2.65	0.685
IL-1β (pg/mL)	16.48^c^	26.06^a^	21.33^b^	2.63	0.005
IL-2 (pg/mL)	183.9^c^	298.6^a^	233.4^b^	22.1	0.006
IL-4 (pg/mL)	10.35^a^	6.68^b^	7.34^b^	1.13	0.026
IL-6 (pg/mL)	111.7^b^	155.9^a^	124.1^b^	9.64	0.017
LPS(EU/L)	15.44^b^	24.91^a^	18.34^b^	2.24	0.013

Abbreviations: CON, control treatment; H_2_S, hydrogen sulfide treatment; LAC, *Lactobacillus* supplement treatment; LPS, lipopolysaccharide; IL, interleukin.

a,b,c means within a row with different letters differed significantly (*P* < 0.05).

### Effects of Lactobacillus Supplementation on Blood Metabolites Under Hydrogen Sulfide Treatment

Metabolomic results were obtained using a filtering method, with a null value threshold of 50% or lower, resulting in the identification of 477 metabolites across all samples. A detailed list of all identified metabolites can be found in supplemental file 1.

**Figure 1:**
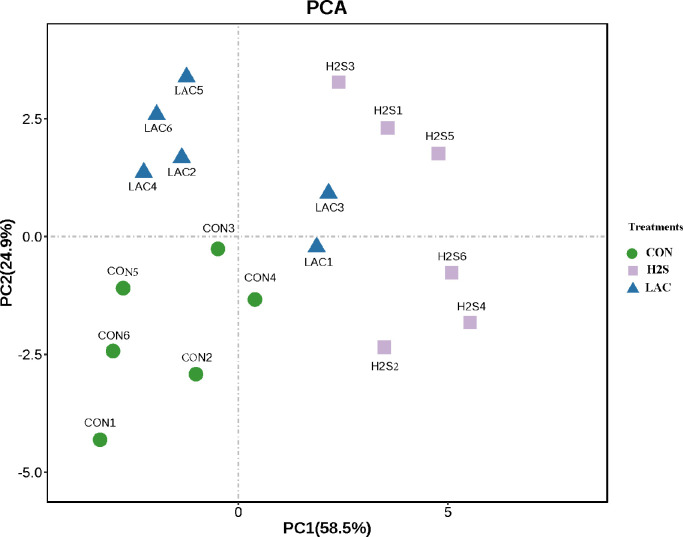
Principal component analysis on serum metabolites between the control and consecutive H_2_S treatment layer hens. Abbreviations: CON, control treatment; H_2_S, hydrogen sulfide treatment; LAC, Lactobacillus supplement treatment.

As Figure 1 shows, PC1 and PC2 accounted for 58.5% and 24.9% of the total variation, respectively. Metabolites in the H_2_S exposure treatment differed significantly from those in the CON and LAC treatments based on PC1 and PC2. Metabolites in the LAC treatment could be differentiated from the CON group by PC1 and PC2.

Differential metabolite analysis was conducted between the H_2_S exposure and CON groups, and between LAC and H_2_S exposure groups, using statistical criteria of fold change > 2, variable importance in projection (**VIP**) > 1, and *P* < 0.05 (Tables 4 and 5). A total of 33 significant differential metabolites, comprising 15 upregulated and 18 downregulated metabolites, were detected between the H_2_S exposure and CON groups. The upregulated metabolites included D-glucopyranuronic acid, deoxyuridine, 3-phenyllactic acid, guaifenesin, indole-3-acetic acid, isoquinoline, cholic acid, mimosine, and others. The down-regulated metabolites mainly consisted of palmitoleic acid, desaminotyrosine, D-fructose, lauric acid, linoleic acid, and oleic acid. Compared with the H_2_S exposure group, after *Lactobacillus* supplementation, the content of aceturic acid, 9-oxodecenoic acid, palmitoleic acid, lauric acid, linoleic acid, oleic acid, and valeric acid significantly increased, while the levels of deoxyuridine, 3-phenyllactic acid, guaifenesin, indole-3-acetic acid, and mimosine significantly decreased.

**Table 4:** Differential analysis on the metabolites between consecutive H_2_S treatment and control treatment (n = 6).

Items		FC	*P*-value	VIP
Up-regulated	D-Glucopyranuronic acid	7.87	0.047	1.573
	deoxyuridine	7.16	0.003	2.702
	3-Phenyllactic acid	5.33	0.033	1.669
	Guaifenesin	4.96	0.035	1.801
	Indole-3-acetic acid	3.77	0.046	2.052
	Isoquinoline	3.54	0.046	2.143
	cholic acid	2.92	0.042	1.516
	Mimosine	2.72	0.025	1.973
	Phenobarbital	2.67	0.049	1.590
	Isoquinoline	2.58	0.036	1.609
	1-Hexadecanoylpyrrolidine	2.64	0.016	1.849
	N-Acetyl-L-leucine	2.59	0.045	1.493
	N-Acetylvaline	2.67	0.047	1.713
	2-Furoic acid	2.44	0.047	1.236
	β-Alanine	2.12	0.027	1.349
Down-regulated	Aceturic acid	0.49	0.024	1.786
	Arabinosylhypoxanthine	0.48	0.022	1.841
	Palmitoleic acid	0.42	0.035	1.881
	Desaminotyrosine	0.42	0.007	2.079
	D-Fructose	0.38	0.002	2.361
	Lauric acid	0.37	0.014	1.979
	Linoleic acid	0.37	0.032	1.886
	Oleic acid	0.33	0.024	1.986
	Levetiracetam	0.32	0.015	1.861
	Juniperic acid	0.31	0.025	1.995
	Glycylglutamine	0.29	0.015	2.010
	Valeric acid	0.27	0.013	2.125
	D-Arabinose	0.26	0.001	2.345
	Tryptophanamide	0.25	0.048	1.681
	Carbendazim	0.18	0.006	2.109
	Methyl bisnorbiotinyl ketone	0.17	0.004	2.139
	Kojic acid	0.16	0.001	2.387
	Miglitol	0.14	0.001	2.556

Abbreviations: FC, fold change; VIP, variable importance in the projection.

**Table 5:** Differential analysis on the metabolites between *Lactobacillus* supplement treatment and consecutive H_2_S treatment.

Items		FC	*P*-value	VIP
Up-regulated	Palmitoleic acid	6.25	0.035	1.880
	Desaminotyrosine	3.70	0.017	2.179
	D-Fructose	3.45	0.012	2.361
	Lauric acid	3.23	0.014	1.279
	Linoleic acid	3.13	0.042	1.486
	Oleic acid	3.03	0.034	1.786
	Levetiracetam	2.70	0.025	1.861
	Juniperic acid	2.70	0.015	1.995
	Glycylglutamine	2.63	0.045	2.101
	Valeric acid	2.38	0.033	2.125
	Kojic acid	2.38	0.041	2.487
	Miglitol	2.25	0.011	2.556
Down-regulated	deoxyuridine	0.47	0.003	1.702
	3-Phenyllactic acid	0.41	0.033	1.269
	Guaifenesin	0.37	0.035	1.401
	Indole-3-acetic acid	0.37	0.046	2.152
	Mimosine	0.27	0.025	1.473
	Phenobarbital	0.20	0.049	1.859
	2-Furoic acid	0.19	0.047	2.236
	β-Alanine	0.14	0.027	1.949

Abbreviations: FC, fold change; VIP, variable importance in the projection.

**Figure 2:**
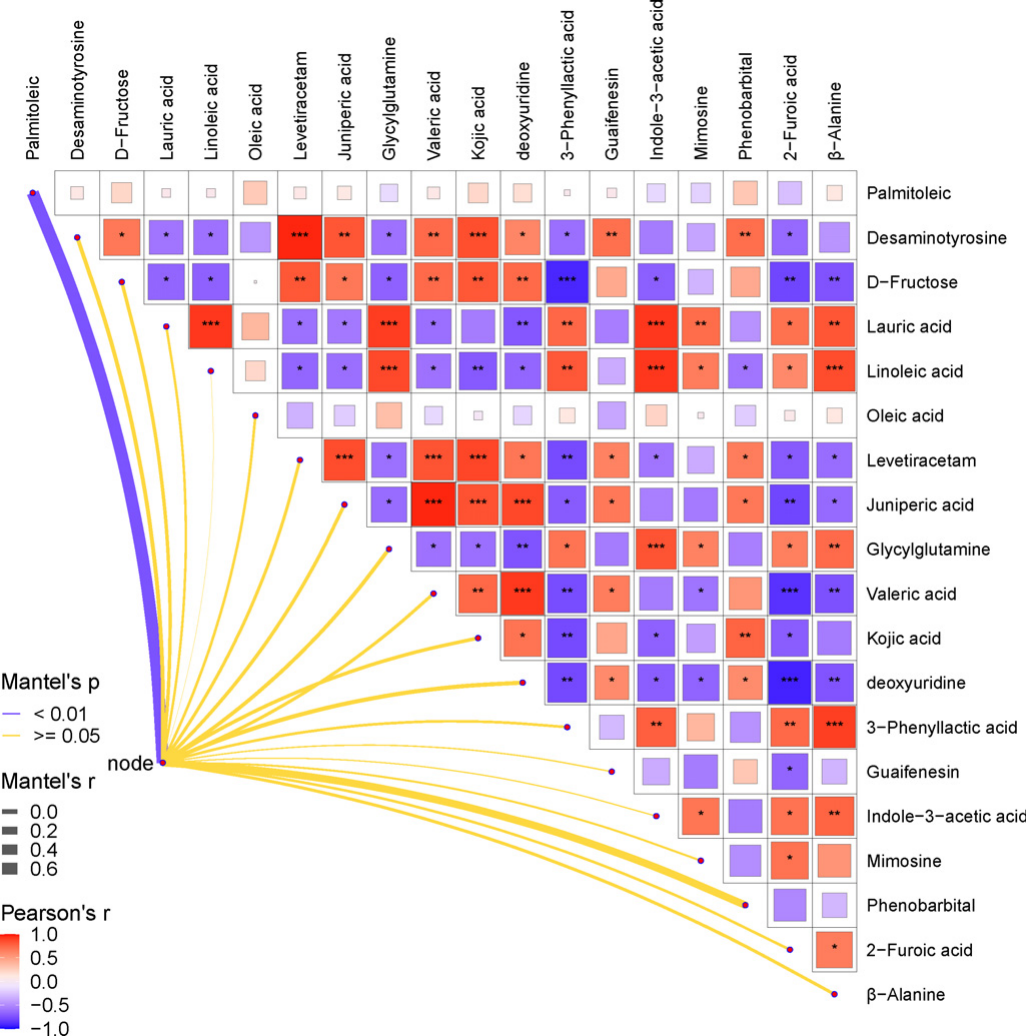
Pearson correlation analysis between *Lactobacillus* and the relative content of blood metabolites. The red color represents a positive correlation while the blue color represents a negative correlation. “*” means a significant correlation (0.55 >|r| > 0.35, *P* < 0.05). “**” means (0.75>|r| > 0.55, *P* < 0.01); “***” means (|r| > 0.75, *P* < 0.001).

We observed a negative correlation between *Lactobacillus* supplementation and palmitoleic acid (Figure 2). However, no significant correlation was observed between *Lactobacillus* supplementation and other metabolites. Intriguingly, palmitoleic acid showed no significant correlation with other metabolites. In addition, desaminotyrosine and D-fructose exhibited a positive interaction with levetiracetam, juniperic acid, valeric acid, kojic acid, and guaifenesin in response to *Lactobacillus* supplementation*.* Conversely, lauric acid and linoleic acid showed a complete inverse correlation with desaminotyrosine or D-fructose.

**Figure 3:**
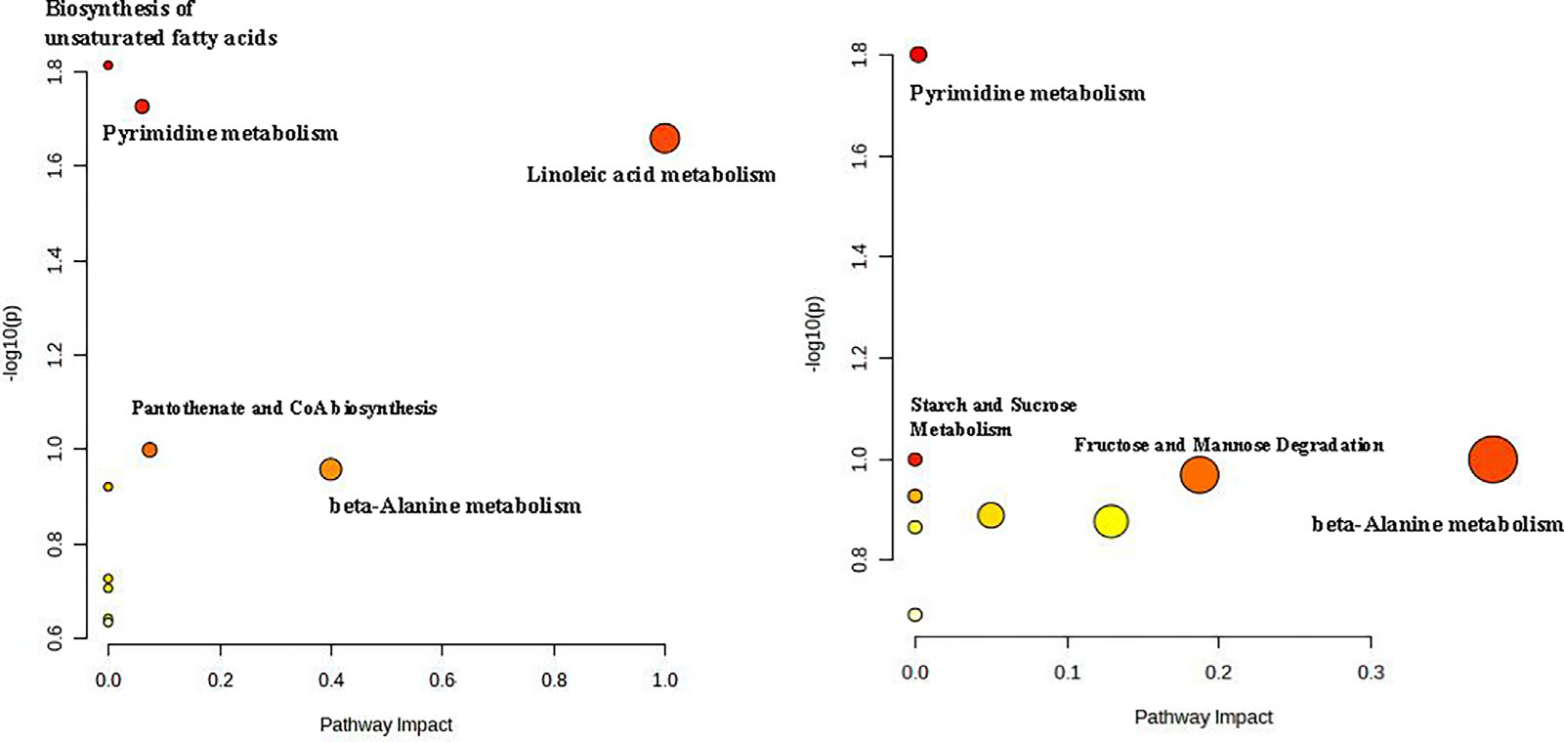
KEGG pathway analysis on differential metabolites between among the treatments. (A) Pathway analysis on differential metabolites between control and consecutive H_2_S treatments. (B) Pathway analysis on the differential metabolites between *Lactobacillus* supplement treatment and consecutive H_2_S treatment.

The pathway analysis between the CON and H_2_S groups (shown in Figure 3) indicated that the enriched pathways were biosynthesis of unsaturated fatty acids, pyrimidine metabolism, linoleic acid metabolism, pantothenate and CoA biosynthesis, and beta-alanine metabolism (Figure 3A). The pathway analysis between the LAC and H_2_S groups revealed pyrimidine metabolism, starch and sucrose metabolism, fructose and mannose degradation, and beta-alanine metabolism as the most impactful effects.

### Effects of Lactobacillus Supplementation on Intestinal Morphology Under Hydrogen Sulfide Treatment

**Figure 4:**
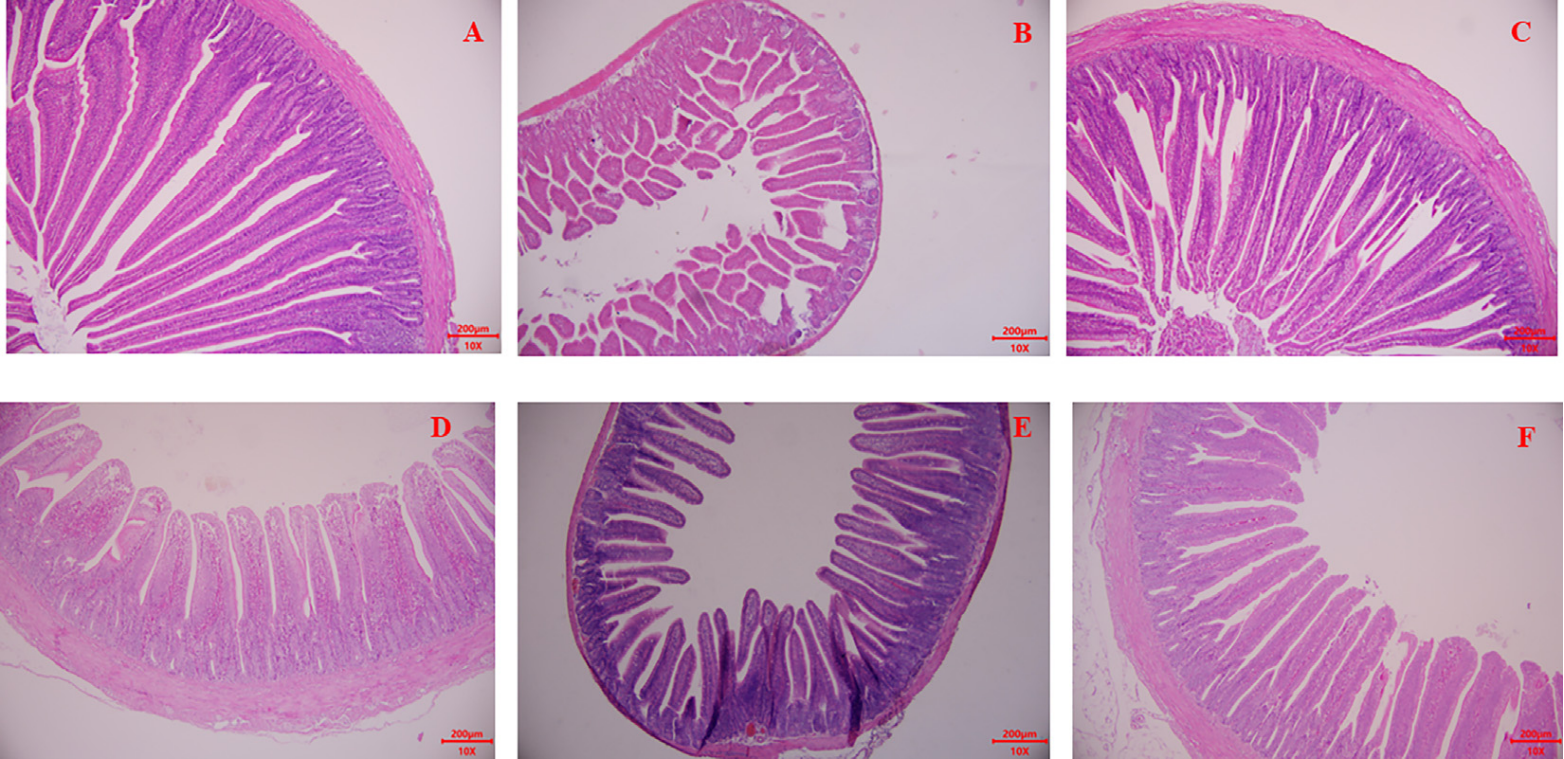
Effects of *Lactobacillus* supplement on ileum and jejunum morphological alternative of broilers under consecutive H_2_S treatment. (A) Paraffin section of jejunum morphology of broilers in control treatment. (B) Paraffin section of jejunum morphology of broilers under H_2_S exposure treatment. (C) Paraffin section of jejunum morphology of broilers received *Lactobacillus* supplement under H_2_S exposure treatment. (D) Paraffin section of ileum morphology of broilers in control treatment. (E) Paraffin section of ileum morphology of broilers under H_2_S exposure treatment. (F) Paraffin section of ileum morphology of broilers received *Lactobacillus* supplement under H_2_S exposure treatment.

The morphology results of the development of ileum and jejunum are presented in Figure 4 and Table 6. In the ileum, the villus height exhibited a significant decrease under H2S exposure compared with the CON group. However, following *Lactobacillus* supplementation, a significant increase in villus height was observed (*P* < 0.05). No significant changes were observed in crypt depth, wall thickness, and villus/crypt ratio of the ileum (*P* > 0.05). Furthermore, no significant alterations were observed in villus height, crypt depth, wall thickness, and villus/crypt ratio in jejunum across all treatments (*P* > 0.05).

**Table 6:** Effects of *Lactobacillus* supplement on ileum and jejunum morphological alternative of broilers under consecutive H_2_S treatment (n = 6).

Items		CON	H_2_S	LAC	SEM	*P*-value
ileum	Villus height	767.4^a^	723.3^b^	751.4^a^	23.26	0.026
	Crypt depth	92.14	87.41	89.78	11.31	0.355
	Thickness	247.3	235.1	246.9	17.67	0.534
	Villus /Crypt	8.33	8.27	8.37	0.142	0.227
jejunum	Villus height	914.4	878.5	897.2	45.1	0.117
	Crypt depth	95.32	92.73	94.01	9.21	0.298
	Thickness	195.9	198.1	196.9	16.32	0.783
	Villus /Crypt	9.59	9.47	9.54	0.46	0.183

Abbreviations: CON, control treatment; H_2_S, hydrogen sulfide treatment; LAC, *Lactobacillus* supplement treatment.

a,b means within a row with different letters differed significantly (*P* < 0.05).

### Effects of Lactobacillus Supplementation on Gut Bacteria Communities Under Hydrogen Sulfide Treatment

A total of 12 phyla and 210 genera were identified using the filtering method, and results are shown in supplemental file 2. Alpha-diversity, which reflects the internal complexity of each treatment, was evaluated, and the results are presented in Table 7. The Shannon and ACE indexes significantly decreased after the H_2_S exposure treatment compared with the CON group (*P* < 0.05). In contrast, the Shannon index significantly increased after *Lactobacillus* supplementation (*P <* 0.05), while no significant changes were observed in the ACE index (*P* > 0.05). No significant alterations were observed in the Simpson and Chao1 indexes across all 3 treatments (*P* > 0.05).

**Table 7:** Effects of *Lactobacillus* supplement on cecal bacterial α-diversity of broilers under consecutive H_2_S treatment of layer hens (n = 6).

Items	CON	H_2_S	LAC	SE	*P*-value
Shannon	4.87^a^	4.56^b^	4.73^a^	0.09	0.305
Simpson	0.93	0.94	0.94	0.01	0.174
ACE	912.7^a^	882.7^b^	889.4^b^	17.7	0.042
Chao1	920.6	897.5	904.4	28.6	0.114

Abbreviations: CON, control treatment; H_2_S, hydrogen sulfide treatment; LAC, Lactobacillus supplement treatment.

a,b means within a row with different letters differed significantly (*P* < 0.05).

**Figure 5:**
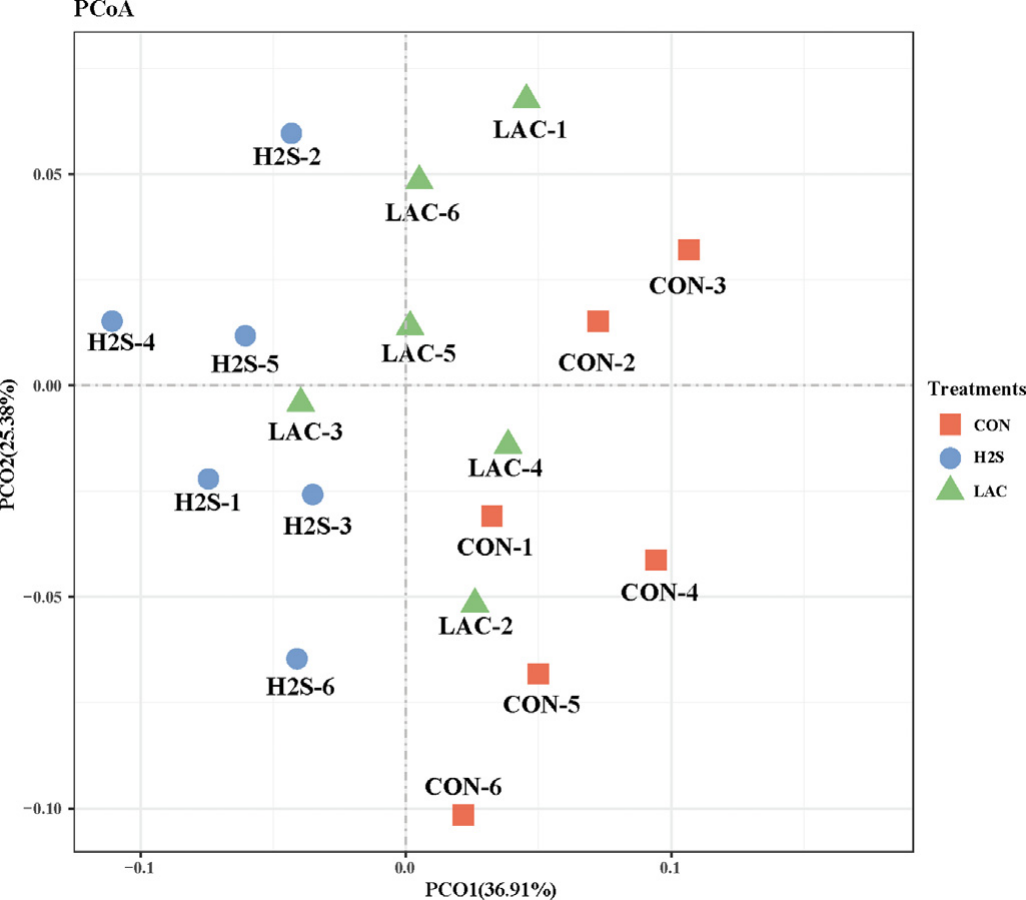
Principal coordinate analysis (PCoA) on community structures of the cecal microbiota after consecutive H_2_S treatment of layer hens. Abbreviations: CON, control treatment; H_2_S, hydrogen sulfide treatment; LAC, Lactobacillus supplement treatment.

Beta-diversity results were visualized through principal coordinates analysis (PCoA; Figure 5). PCoA axes 1 and 2 accounted for 36.91% and 25.38% of the total variation, respectively. Microbial communities showed a significant separation between the CON and H_2_S groups along PCo1. The members of LAC group, except LAC 3, were clearly separated from the H_2_S exposure group along both PCo1 and PCo2. Microbial communities did not show clear separation between the CON and LAC groups.

The impacts of H_2_S exposure and *Lactobacillus* supplementation on the relative abundances of cecal bacteria are presented in Table 8. *Faecalibacterium, Parabacteroides, Ruminococcaceae, Prevotella*, and *Ruminococcus* constituted the 5 most abundant genera, accounting for more than 60% of bacterial content in either treatment. Specifically, H_2_S treatment significantly increased the relative abundances of *Faecalibacterium* and *Ruminococcaceae*, but remarkably decreased those of *Prevotella, Lactobacillus, Bifidobacterium, Clostridium*, and *Campylobacter* compared with the CON group (*P* < 0.05). In contrast, *Lactobacillus* supplementation significantly decreased the relative abundance of *Faecalibacterium*, and induced significant increases in the relative abundances of *Lactobacillus, Bifidobacterium, Clostridium*, and *Butyricimonas* (*P* < 0.05). No other significant changes were detected in the relative abundances of other genera among all treatments (*P* > 0.05).

**Table 8:** Effects of *Lactobacillus* supplement on the relative abundances of cecal bacterial in the level of genera of broilers under consecutive H_2_S treatment of layer hens (n = 6).

Genera (%)	CON	H_2_S	LAC	SEM	*P*-value
*Faecalibacterium*	13.97^b^	16.23^a^	14.21^b^	1.17	0.027
*Parabacteroides*	13.01	11.67	12.52	2.14	0.141
*Ruminococcaceae*	10.06^b^	12.13^a^	11.93^a^	0.73	0.031
*Prevotella*	14.01^a^	10.77^b^	12.83^a^^b^	1.06	0.023
*Alistipes*	8.68	9.88	9.65	0.97	0.222
*Ruminococcus*	10.13	9.48	10.83	1.12	0.478
*Oscillospira*	6.42	6.08	5.66	1.05	0.098
*Sutterella*	3.65	2.49	2.88	0.96	0.214
*Phascolarctobacterium*	4.11	3.64	4.64	0.76	0.231
*Helicobacter*	1.34	1.26	1.31	1.22	0.478
*Butyricimonas*	1.28	1.37	1.22	0.85	0.398
*Coprococcus*	1.36	1.30	1.28	0.59	0.171
*Lactobacillus*	0.66^b^	0.38^c^	1.03^a^	0.16	0.014
*Methanobrevibacter*	0.14	0.27	0.14	0.07	0.064
*Bifidobacterium*	0.19^a^	0.13^b^	0.23^a^	0.02	0.021
*Clostridium*	0.21^a^	0.11^b^	0.24^a^	0.04	0.033
*Campylobacter*	0.31^a^	0.15^b^	0.29^a^	0.07	0.013
*Streptococcus*	0.04	0.07	0.05	0.02	0.108
others	9.42	9.73	9.07	0.48	0.323

Abbreviations: CON, control treatment; H_2_S, hydrogen sulfide treatment; LAC, Lactobacillus supplement treatment.

a,b,c means within a row with different letters differed significantly (*P* < 0.05).

### Correlation Analysis between Immunity Parameters and Bacterial Communities

**Figure 6:**
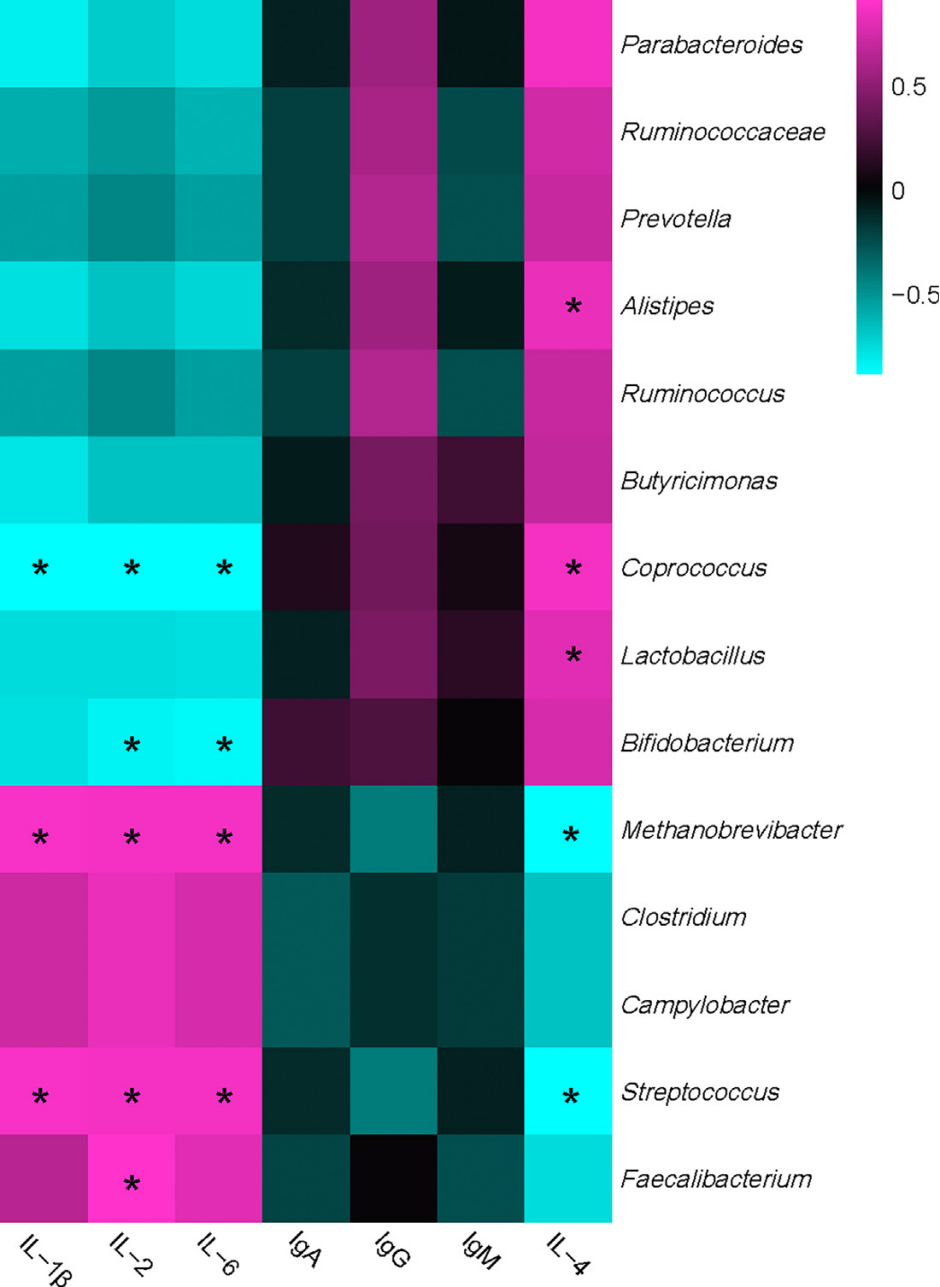
Correlation analyses between abundances of cecal bacteria and host serum immunity-related parameters. The red color represents positive correlation while the bule color represents a negative correlation. “*” means a significant correlation (|r| > 0.55, *P* < 0.05).

As shown in Figure 6, bacterial communities were divided into 2 distinct groups based on their correlations with immunity-related parameters. The first group primarily consisted of *Ruminococcaceae, Alistipes, Coprococcus, Butyricimonas, Lactobacillus*, and *Bifidobacterium*, which negatively correlated with IL-1β, IL-2, and IL-6, but positively correlated with IgG and IL-4. The second group consisted of *Methanobrevibacter, Faecalibacterium, Clostridium, Campylobacter*, and *streptococcus*, showed a completed inverse correlation with the immunity-related parameters compared with the first group. Specifically, *Coprococcus*, and *Bifidobacterium* showed a significantly negative correlation with IL-2 and IL-6, whereas, *Methanobrevibacter* and *streptococcus* positively correlated with IL-1β, IL-2, and IL-6, respectively. *Coprococcus* and *Lactobacillus* showed remarkably positive correlation with IL-4, but *Methanobrevibacter* and *streptococcus* presented a complete reverse correlation with IL-4. In addition, *Faecalibacterium* presented a positive correlation with IL-2, while *Alistipes* showed positive correlation with IL-4.

## DISCUSSION

*Lactobacillus* has been widely studied and recognized as a beneficial probiotic. It has been shown to have various positive effects, including the prevention of intestinal infectious diseases, promotion of overall health, and improvement of productive performance and product quality of poultry (Spivey et al., 2014; Shokryazdan et al., 2017). These beneficial effects can be attributed to the following factors.

Generally, inhalation of atmospheric H_2_S can trigger inflammatory and immune responses, leading to an increase in interleukin levels (Wang et al., 2018; Hu et al., 2019). Similar results like the higher IL-1β, IL-2, and IL-6 after inhalation of H_2_S are also observed in our study, and further supported the inflammatory-inducing effect of H_2_S. *Lactobacillus* supplementation significantly suppressed these elevated inflammatory responses, consistent with the findings of Li et al. (2022),who highlighted the role of probiotics in reducing inflammatory responses and mitigating excessive inflammation-induced damage in infected broilers.

One of the key reasons why *Lactobacillus* can bolster host immunity may be associated with the tolerance of mucosal immunology to probiotic bacteria invasion (Shokryazdan et al., 2017). Upon successful colonization in the cecal portion, *Lactobacillus* efficiently metabolizes undigested nutrients, producing numerous beneficial secondary metabolites such as flavonoids, bile acids, and phenolic acid compounds (Sentürk et al., 2020; Wang and Yao, 2021). These metabolites possess various immunomodulatory properties and significantly improve physiological immunity (Peluso et al., 2015; Fiorucci et al., 2018). In addition, *Lactobacillus* is positively associated with increased butyrate content (Berni Canani et al., 2016), which serves as a crucial energy source for gut enterocytes, effectively reduces inflammatory responses and gastrointestinal pathogens, and helps regulate gut bacterial ecosystem. *Lactobacillus* may help alleviate the effects of H_2_S exposure by promoting butyrate generation. Furthermore, *Lactobacillus* has also been shown to interact positively with serum IgA to enhance its functions (Ashraf and Shah, 2014; Lai et al., 2019). This interaction may explain the significant changes in IgA levels and the subsequent improvement in immunity.

Our study supports the findings of Gong et al. (2020) that a higher abundance of cecal *Lactobacillus* improves intestinal morphology. This was evident in our observations through the significant increase of ileum villus height following *Lactobacillus* supplementation. A healthy gastrointestinal tract is characterized by a well-structured morphology and a stable microbial environment. Probiotics, including *Lactobacillus*, play important roles in strengthening the intestinal mucus layer and promoting villus development (Luo et al., 2018; Engevik et al., 2019). The promoting effects of *Lactobacillus* might be attributed to the potential increase in butyrate content. Butyrate is particularly important as an energy source and plays a role in modulating the expression of certain epithelial genes for epithelial development in gut (Chen et al., 2018). In the present research, the significant increase in *Butyricimonas*, a key butyrate-generating bacterium, after *Lactobacillus* supplementation indicates a potential enhancement of epithelial development. Higher villus height and a higher villus/crypt ratio contribute to improved nutrient absorption from feed in the intestine (Wang et al., 2021). As a result, the BWG of broilers significantly increased after *Lactobacillus* supplementation.

In addition, broiler physiological resistance to metabolic disorder, and maintenance on homeostasis were also enhanced after *Lactobacillus* supplement, especially the enhancement of starch and sucrose metabolism, as well as fructose and mannose degradation metabolism which were in line with Wang et al. (2021) and Forte et al. (2018), may also contribute to the increase in BWG. Starch is a major nutrient in poultry diets, providing a significant amount of energy for bodily metabolism and development (Svihus, 2014). As an easily degradable carbohydrate, starch is hydrolyzed into disaccharides, and further into glucose and fructose. The increase in fructose and mannose degradation metabolism following *Lactobacillus* supplementation may contribute to the generation of more energy for metabolism and overall body development. This increased availability of energy sources likely supports the observed increase in BWG following *Lactobacillus* supplementation under H_2_S exposure.

Moreover, the crosstalk between the host and the associated bacteria plays a crucial role in providing resistance against hydrogen sulfide exposure by effectively shaping the gastrointestinal tract, enhancing productive performance, and improving immunity (Jackrel et al., 2020). Intestinal epithelial cells (**IECs**) act as functional barriers to protect the intestinal mucosa from pathogenic microorganisms and facilitate nutrient absorption into circulation through various functional proteins (Xie et al., 2019). Previous study suggested the stability of epithelial barrier was easily perturbed by activated pathogenic bacteria through secreting LPS that cause inflammation in the body, and leading to the increase of IL-1β, IL-2, and IL-6 (Ohno et al., 2020). Probiotics supplement (e.g., *Butyricimonas, Lactobacillus*, and *Bifidobacterium*), re-shaped the composition in gut microbiota, reduced pathogenic bacteria abundances and LPS secretion, and thereafter alleviated the inflammatory responses (Zhou et al., 2022). Besides, secondary metabolites that secreted by probiotics such as flavonoids, exert anti-inflammatory effects through blockade of NF-κB, inhibition of pro-inflammatory cytokines production including the IL-1β, IL-2, IL-6, and TNF-α, and reduction of reactive oxygen species (Martínez et al., 2019). In our study, pro-inflammatory cytokines also decreased after *Lactobacillus* treatment, these findings further supported the anti-inflammatory effects of *Lactobacillus.* Besides, the intestinal microbiota and the host co-evolve, establishing a stable intestinal micro-environment to provide the host with diverse bio-functions, such as the digestion of complex dietary carbohydrates and maintenance of intestinal homeostasis (Forte et al., 2018; Valdes et al., 2018). Therefore, *Lactobacillus* supplementation effectively modulates the composition of intestinal microbial communities, closely interacts with the host immune systems to enhance overall immunity, and ultimately promotes the growth performance of broilers exposed to H_2_S.

**Figure 7:**
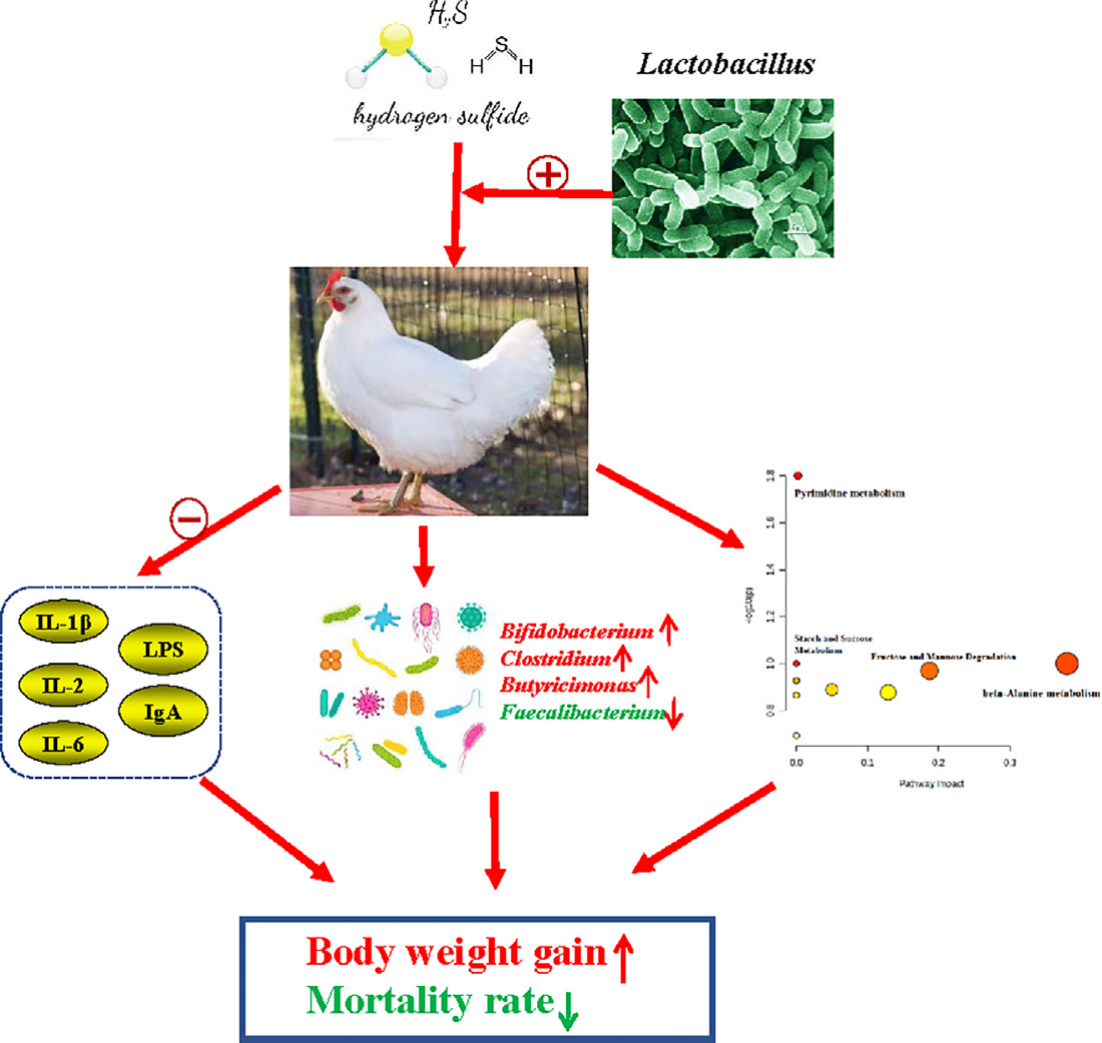
Underlying mechanism of *Lactobacillus* supplement on productive performances of broilers under consecutive H_2_S treatment. The red color indicated an increment, while the green color represented a decrease.

In summary, *Lactobacillus* supplementation effectively improved host immunity and alleviated inflammatory responses by reducing serum levels of IL-1β, IL-2, IL-6, LPS, and IgA. Moreover, *Lactobacillus* supplementation reshaped the composition of the intestinal microbiota, leading to a decrease in *Faecalibacterium* and an increase in *Bifidobacterium, Clostridium*, and *Butyricimonas*, which further induced changes in blood metabolites and enhanced nutritional degradability. Consequently, these effects resulted in improved body weight gain and reduced mortality rates (Figure 7).

## DISCLOSURES

Animal care and procedures followed The Chinese Guidelines for Animal Welfare, which was approved by the Animal Care and Use Committee of Jiangxi Agricultural University, with the approval number JXAULL-20230126.

ALL authors declare that the research was conducted in the absence of any commercial or financial relationships that could be construed as a potential conflict of interest.
